# Age-specific efficacy of biologics in severe asthma: a pediatric perspective

**DOI:** 10.3389/falgy.2026.1777666

**Published:** 2026-05-15

**Authors:** Beatrice Andrenacci, Francesca Calciano, Barbara Madini, Alessia Rocchi, Daniele Giovanni Ghiglioni, Laura Maffeis, Maria Francesca Patria, Amelia Licari, Gian Luigi Marseglia

**Affiliations:** 1 Fondazione IRCCS Ca’ Granda, Ospedale Maggiore Policlinico, SC Pediatria Pneumoinfettivologia, Milan, Italy; 2 Università Degli Studi di Milano, Milan, Italy; 3 Fondazione IRCCS Ca’ Granda, Ospedale Maggiore Policlinico, SC Pediatria Immunoreumatologia, Milan, Italy; 4 Pediatric Unit, Department of Clinical, Surgical, Diagnostic and Pediatric Sciences, University of Pavia, Pavia, Italy; 5 Pediatric Clinic, Fondazione IRCCS Policlinico, Pavia, Italy

**Keywords:** age-specific efficacy, biologic therapies, dupilumab, mepolizumab, omalizumab, pediatric precision medicine, pediatric severe asthma, tezepelumab

## Abstract

Severe pediatric asthma is a rare yet burdensome disease, often unresponsive to conventional anti-asthmatic treatments and associated with significant morbidity, impaired lung development, and high healthcare use. Over the last two decades, biologic therapies have deeply changed the treatment of severe asthma, with both clinical and functional efficacy and a favorable safety profile. However, most studies and clinical trials on the field have focused on adults, leaving a relevant gap in pediatric-specific evidence. Since pediatric responses may deeply diverge from adults due to age-specific immunological and physiological factors, this narrative review aims to summarize current pediatric-specific evidence on the efficacy and safety of Europe-licensed anti-asthmatic biologics for pediatric severe asthma, highlighting key differences from adult data and discussing emerging challenges and future research directions such preventive use of biologics, innovative delivery methods, combination treatments, standardized definitions of asthma remission and development of pediatric-specific outcome measures, ultimately leading to individualized therapy.

## 1 Introduction

Asthma is the most common chronic respiratory disease in children, affecting approximately 10% of children and adolescents worldwide (1). Although the majority of cases can be controlled with inhaled corticosteroids (ICS), a small but significant proportion of pediatric patients (2) develop severe asthma, defined as disease that remains uncontrolled despite maximal optimized treatment, or that worsens when such treatment is stepped down (3). European pediatric data indicate that severe asthma affects 3% of children in Northern Europe and up to 8% in Mediterranean countries, with differences depending on the severe asthma definition used (2).

Despite its rarity, severe asthma accounts for up to half of the direct and indirect costs of pediatric asthma care (1). Children and adolescents with severe asthma experience increased morbidity and even mortality, reduced personal and familiar quality of life (QoL) and worse lung function trajectories, with higher risk of developing progressive airway remodeling and fixed airflow obstruction (4).

In the effort to standardize definitions, the American Thoracic Society (ATS) and the European Respiratory Society (ERS) have proposed and distinguished three main categories among severe asthma: *difficult-to-treat asthma*, *refractory difficult asthma*, and *severe therapy-resistant asthma* (STRA), depending on the presence of concomitant addressable or not addressable “treatable traits” (5). In STRA, asthma severity depends only on inflammatory characteristics and benefits the most from tailored, personalized treatments.

Asthma pathogenesis derives from a complex interplay among genetics, innate and adaptive immunity, microbiome and exposure to exogenous and endogenous *noxae* (collectively named “exposome”) with the active involvement of both inflammatory mediators and airway structural cells (6, 7). Therefore, asthma is now recognized as an umbrella term encompassing different *phenotypes* (observable clinical characteristics) and *endotypes* (underlying molecular pathways) (7). Notably, asthma phenotypes and endotypes have unique features in pediatrics, reflecting age-dependent immaturity of immune and respiratory systems, different exposomes and diverse associated comorbidities compared to adult asthmatics (8). Currently, the most common classification of pediatric severe asthma endotypes covers five different airway inflammatory entities, which can be assessed on bronchoalveolar lavage (BAL): eosinophilic allergic asthma, eosinophilic non allergic asthma, neutrophilic asthma, paucigranulocytic asthma and mixed asthma (9).

Eosinophilic allergic asthma is the most common pediatric STRA endotype and is characterized by allergen-driven type 2 (T2-high) inflammation mediated by T helper 2 (TH2) cytokines, including interleukin (IL)-4, IL-5, and IL-13 (10). Eosinophilic non-allergic asthma is another T2-high endotype triggered by epithelial-derived “alarmins” (TSLP, IL-25, IL-33) that activate innate lymphoid cells type 2 (ILC2) in response to epithelial airway damage, leading to type 2 cytokine production (11). Neutrophilic and paucigranulocytic asthma are T2-low endotypes, characterized by low Th2 cytokines and higher expression of IFN-*γ*, IL-8, IL-17, and IL-22, reflecting Th1/Th17-driven inflammation. These forms are less common in pediatrics, even in severe asthma, and are typically more steroid-resistant. Pediatric neutrophilic asthma remains controversial, as airway neutrophilia may reflect chronic inhaled corticosteroid use or intercurrent infections and may even play a protective role depending on airway micro-compartmentalization (9). Paucigranulocytic asthma is rare and is characterized by minimal airway inflammation on BAL, with disease mechanisms driven primarily by airway structural cells and neuronal pathways (9). Finally, mixed asthma combines features of multiple endotypes and is usually a steroid-resistant T2-high/T2-low severe phenotype (9).

When standard therapies fail, oral corticosteroids (OCS) may be required, which are poorly tolerated in children due to significant short- and long-term adverse effects, even at low doses (12). Over the past 25 years, biologic therapies have emerged as targeted alternatives for specific severe asthma endotypes. Biological treatments for severe asthma mainly consist in humanized monoclonal antibodies, uniquely targeting specific mediators of airway inflammation. Their introduction has dramatically improved asthma outcomes, particularly in patients with T2-high endotypes, leading to better symptom control, fewer exacerbations, reduced ICS and OCS exposure, and improved QoL for both children and caregivers (13), with long-term (up to 7 years of treatment) good pediatric safety (14). In Europe, four biologics are currently licensed for pediatric asthma: omalizumab, mepolizumab, dupilumab, and - most recently – tezepelumab.

A large network meta-analysis on 16,350 subjects from 48 randomized controlled trials (RCTs) reported that anti-asthmatic biologics collectively reduce asthma exacerbations by 44% and hospitalizations by 60% and improve forced expiratory volume in 1 s (FEV₁) by an average of +110 mL, alongside meaningful gains in asthma control, while maintaining favorable safety profiles (15). However, as most phase 3 trials on biologics were conducted primarily in adults, pediatric evidence data remain limited and are largely derived from small subgroups, *post hoc* analyses, or extrapolated thresholds, with a lack of head-to-head pediatric comparison studies (16). As children differ in their immunopathogenesis, eosinophilic responses, and patterns of steroid responsiveness, this raises concerns about directly applying adult trial results to pediatric populations (17), which cannot be assumed to mirror adult outcomes.

Therefore, the present narrative review aims to assess the most recent, pediatric-specific evidence of efficacy and safety of currently EU-licensed biologics for pediatric severe asthma, while highlighting current gaps and future research directions. Other biologics such as benralizumab, reslizumab and depemokimab were not considered, since they are not yet approved for pediatric asthma in the European Union and therefore fall outside the scope of the present review.

## 2 Methods

For this narrative review, a comprehensive literature search was conducted in PubMed to identify relevant publications on biologic therapies for pediatric severe asthma. The search was limited to full-text, English-language articles published between January 2020 and May 2025. Only studies including participants in the pediatric age range were considered. The search strategy combined the following keywords: “*omalizumab OR dupilumab OR mepolizumab OR tezepelumab AND asthma”* and “*biologic efficacy OR biologic safety AND asthma”*.

Two reviewers independently screened titles, abstracts, and subsequently full-text articles. Eligible study types included RCTs, real-world studies, narrative and systematic reviews, and meta-analyses.

The initial search yielded 89 studies. After removal of 10 duplicates, 19 non-pediatric studies, and 3 articles not addressing efficacy or safety outcomes, a total of 47 studies were included in the final analysis. In addition, critical phase 3 RCTs including pediatric subgroups and landmark articles in the field were retained for completeness. Reference lists of key papers were also hand-searched to identify additional relevant publications. Since the narrative nature of the review, no PRISMA flowchart or formal risk-of-bias assessment was performed.

## 3 Omalizumab

Omalizumab is a humanized monoclonal antibody targeting immunoglobulin E (IgE). By binding circulating IgE and downregulating Fc*ε*RI expression, omalizumab reduces mast cell and basophil activation (18) and therefore eosinophilic inflammation, concurrently enhancing antiviral responses through IFN-α modulation. In Europe, omalizumab is approved for children ≥6 years with severe uncontrolled allergic asthma (serum IgE levels 30–1,500 IU/mL) and sensitization to at least one perennial aeroallergen (19). Treatment is administered subcutaneously every 2–4 weeks, with dosing adjusted to baseline IgE and body weight. Omalizumab is licensed also for chronic spontaneous urticaria in subjects ≥12 years (19). As for other biologics, helminth infection should be excluded and possibly treated before starting the treatment.

As the first biologic licensed for pediatric severe asthma, omalizumab has the most extensive pediatric evidence among all biologics. The main double-blind, placebo-controlled RCTs involving also pediatric patients were the Investigation of Omalizumab in Severe Asthma Treatment (**INNOVATE)** (20) the Evaluating Clinical Effectiveness of Omalizumab in Severe Asthma (**EXTRA)** (21) and the Inner-City Anti-IgE Therapy for Asthma (**ICATA)** (22) (Table 1).

**Table 1:** Characteristics and results of main phase-3 studies on omalizumab involving pediatric subjects.

Trial	Population	Pediatric subjects/total	Dosing	Treatment duration (weeks)	Efficacy in pediatrics
INNOVATE	Adolescents (≥12 y) + adults	39/419	SC according to IgE and weight, Q2W or Q4W	28	Significantly reduced severe ER; Improvement in QoL, symptoms and lung function (no pediatric-specific results)
EXTRA	Adolescents (≥12 y) + adults	60/850	SC according to IgE and weight, Q2W or Q4W	48	Significantly reduced ER, especially if FeNO≥20 ppb and Eo ≥ 260cells/μL (no pediatric-specific results); Improvement of FEV₁, ACQ and ACT (n.s.)
ICATA	6–20 y	352/419	SC according to IgE and weight, Q2W or Q4W	60	Significantly reduced hospitalizations and ER during spring and autumn, especially in children 6–11 years; ICS-sparing effect

ACQ, Asthma Control Questionnaire; ACT, asthma control test; Eo, eosinophils; ER, exacerbation rate; FeNO, Fractional exhaled Nitric Oxide; FEV₁, forced expiratory volume in 1 s; n.s., not significant; ICS, inhaled corticosteroids; IgE, immunoglobulin E; OCS, oral corticosteroids; ppb, parts per billion Q2W or Q4W, every 2 or 4 weeks; QoL, quality of life; SC, subcutaneous; y, years.

In the INNOVATE (*n* = 419; 39 adolescents) and EXTRA (*n* = 850; 60 adolescents) trials omalizumab proved to significantly reduce severe exacerbations while improving QoL (20, 21). In the EXTRA trial, greater benefit was observed in children with elevated (≥20 ppb) Fractional exhaled Nitric Oxide (FeNO) or blood eosinophils (≥260 cells/µL). However, limited pediatric sample sizes precluded age-specific conclusions. The ICATA trial, specifically designed for younger populations, provided robust pediatric evidence, proving a reduction of asthma symptoms, asthma attacks and asthma-related health-care utilization with a significant ICS-sparing effect (22). Interestingly, *post hoc* analyses revealed that omalizumab almost abolished the seasonal peaks of asthma attacks typically seen in spring and autumn (23).

Lately, more recent pediatric-specific reviews and real-world studies have confirmed the efficacy of omalizumab in reducing asthma exacerbations and ICS requirements, even in school-aged children. In a double-blind randomized controlled trial (RCT), Milgrom et al. studied 334 children aged 6–12 years treated for 28 weeks with omalizumab or placebo in addition to standard therapy, demonstrating a significant ICS-sparing effect after 16 weeks and a complete discontinuation of ICS for 55% omalizumab children by week 28 (24). Omalizumab was also associated with fewer asthma exacerbations and school absences, although no significant lung function improvements were observed (24). Consistent results were reported in a subsequent RCT involving 626 children aged 6–12 years, which showed fewer severe asthma exacerbations at both 24 and 52 weeks (25). These findings are in line with the results by Chipps et al., whose systematic review confirmed improvements in asthma symptoms, exacerbation rates, unscheduled healthcare visits and ICS dose reduction under omalizumab, with sustained suppression of FeNO despite steroid tapering (26). A 2018 pediatric review further demonstrated a significant reduction in asthma exacerbations after one year of omalizumab treatment, along with improved asthma control (GINA, Asthma Control Questionnaire - ACQ), quality of life (Pediatric Asthma Quality of Life Questionnaire - PAQLQ), reduced healthcare resource utilization and marked steroid-sparing effects (27). Cost-effectiveness was greatest in children with frequent exacerbations, atopic comorbidities, impaired quality of life, and good bronchodilator reversibility (27). More recently, a pediatric-specific meta-analysis of seven RCTs (*n* = 1,700 aged 6–18 years) confirmed that omalizumab significantly reduces severe asthma exacerbations (defined as either requiring OCS for ≥3 days or doubling of ICS dose) both at 24 and 52 weeks (28). As regards long-term efficacy, pediatric-specific data indicate that improvements in FEV₁ were maintained throughout the first year of treatment, while reductions in asthma exacerbations and asthma-related hospitalizations were sustained for up to two years. However, no additional improvements were observed in symptoms, lung function, or inhaled corticosteroid dosing beyond these time points (29). Notably, long-term efficacy in pediatric populations appears to be independent of ethnicity (30).

Intriguingly, recent studies highlighted that the omalizumab suppression of IgE-driven inflammation may result in less TGF-*β*-mediated fibroblast activation, therefore leading to reticular basement membrane (RBM) thickness reduction (31, 32). This especially applies in the presence of higher IgE and greater eosinophil counts, paving the way for the potential use of omalizumab in airway remodelling from its very beginning in early childhood (8).

As concerns omalizumab safety, results have been consistent across pediatric studies, with headache as the most frequently reported adverse event, followed by fever (particularly in younger children), injection-site reactions (more commonly observed in adolescents), and rhinopharyngitis (33). Anaphylaxis is a rare (0.1–0.2%) but potentially severe adverse event, occasionally linked to the presence of the polyethylene glycol (PEG) excipient (34). The incidence of omalizumab-related adverse events appears to be highest during the first month of treatment and to decline progressively thereafter (34). Accordingly, the European Academy of Allergy and Clinical Immunology (EAACI) recommend at least 60 min of inpatient observation after the first three doses of biologics (35). However, continued long-term monitoring is preferrable, since adverse events may also occur beyond one year of treatment (34). As concerns real-life studies, a 4-year prospective study of 23 children (6–18 years) with severe asthma or chronic spontaneous urticaria confirmed long-term omalizumab safety, with no anaphylaxis, serious adverse events, or treatment discontinuations due to side effects (36).

Finally, data on treatment withdrawal seem to suggest durable benefit even after omalizumab discontinuation. A French nationwide real-life study of 2,453 children (median omalizumab duration: 53.7 months) showed a sustained reduction in asthma hospitalizations and OCS use within two years of discontinuation, with 33% children maintaining good asthma control even three years after (37). These findings are consistent with smaller prospective and retrospective real-life pediatric studies which described sustained asthma control, stable lung function, and low exacerbation rates for up to 2 years post-discontinuation, with relatively few relapses (38–40). Intriguingly, the relapses seem to correlate with eosinophil rebound, suggesting post-discontinuation eosinophil monitoring for the early detection of potential clinical deterioration (39, 40). However, available pediatric data on biologic withdrawal mostly derive from observational cohorts without a control group. In the absence of a comparator arm, data cannot properly differentiate potential long-term benefits after omalizumab withdrawal from expected fluctuations in asthma natural history over time. Therefore, current withdrawal results should be interpreted with caution.

## 4 Dupilumab

Dupilumab is a fully humanized monoclonal antibody targeting the *α*-subunit of the interleukin-4 receptor (IL-4R*α*). By blocking IL-4R*α*, the drug simultaneously inhibits IL-4 and IL-13 signaling, involved in IgE class-switching, Th2 cell differentiation, mucus hypersecretion and airway hyperresponsiveness (18).

Currently, dupilumab is licensed in Europe for children ≥6 years with severe, uncontrolled T2-high asthma (blood eosinophils ≥150 cells/µL and/or FeNO≥25 ppb), regardless of allergic sensitization. It is administered subcutaneously every two weeks, at an initial dose of 400 mg (<60 kg) or 600 mg (≥60 kg), followed by 200 mg or 300 mg, respectively (41). Beyond asthma, pediatric approvals include severe atopic dermatitis (≥6 months), eosinophilic esophagitis (≥1 year), and chronic rhinosinusitis with nasal polyps (≥12 years).

The efficacy and safety of dupilumab in pediatric asthma have been assessed in two main phase 3 RCTs and two subsequent extension studies (Table 2).

**Table 2:** Characteristics and results of main phase-3 studies on dupilumab involving pediatric subjects.

Trial	Population	Pediatric subjects/total	Dosing	Treatment duration (weeks)	Efficacy in Pediatrics
QUEST	Adolescents (≥12 y) + adults	107/1,902	SC 200 mg (loading 400 mg) or 300 mg (loading 600 mg) Q2W	52	Increased FEV₁ (especially in T2-high asthma); Reduced AER (n.s.)
VOYAGE	Children (6–11 y)	408/408	SC 100 mg (<30 kg) or 200 mg (≥30 kg) Q2W	52	Reduced AER; Improved FEV₁, FEV₁/FVC and FVC; Increased ACQ-7
EXCURSION	Children (6–11 y)	365/365	SC 100 mg (<30 kg) or 200 mg (≥30 kg) Q2W	52	Reduced AER; Lower OCS use; Lower FeNO, IgE and blood Eo; Better FEV₁
TRAVERSE	Adolescents (≥12 y) + adults	89/2,282	SC 300 mg Q2W	144	Reduced AER (no pediatric-specific results); Better ACQ and AQLQ: Lower Eo and IgE

ACQ, Asthma Control Questionnaire; AER, annualized exacerbation rate; AQLQ, Asthma Quality of Life Questionnaire; Eo, eosinophils; FeNO, Fractional exhaled Nitric Oxide; FEV₁, forced expiratory volume in 1 s; FVC, forced vital capacity; n.s., not significant; IgE, immunoglobulin E; OCS, oral corticosteroids; Q2W, every 2 weeks; SC, subcutaneous; T2, type 2; y, years.

The **LIBERTY ASTHMA QUEST** (*n* = 1,902, 107 adolescents) proved a significant FEV₁ improvement in adolescents after 12 weeks of dupilumab treatment, with no significant reduction of asthma attacks (42). Similarly, the **LIBERTY ASTHMA VOYAGE** trial (*n* = 408; aged 6–11 years), the first fully pediatric phase 3 RCT of dupilumab, demonstrated FEV₁ improvements as early as two weeks after treatment initiation, which were sustained through 52 weeks (43). The study also proved a reduction of annualized exacerbation rates (AERs) irrespective of allergy status, whereas ACQ-7 improvement was more remarkable in children with allergic asthma (44). *post-hoc* analysis proved that all the VOYAGE results were most remarkable in the case of higher eosinophilia (blood eosinophils ≥300–500/µL) and that baseline eosinophilia does not correlate with the risk of treatment-induced hypereosinophilia (45, 46). Therefore, eosinophils monitoring after starting dupilumab is unnecessary unless symptomatic.

Lately, the **EXCURSION trial**, an extension study of VOYAGE (*n* = 365, aged 6–11 years), confirmed a sustained reduction of AERs and OCS use and an improvement of FEV₁ values, FeNO, eosinophils and IgE levels over additional 52 weeks of treatment, with long-term safety (47). Finally, the **TRAVERSE study,** an open-label extension of both QUEST and VENTURE trials (*n* = 2,282, 89 adolescents), confirmed the long-term safety of dupilumab up to three years of treatment, with both sustained reduction of asthma attacks and improvement of FEV₁ (48, 49). Even more recently, a 2025 systematic review (50) pooling 408 children (6–11 years) from VOYAGE and 134 adolescents (12–17 years) from the Simpson trial (which was primarily focused on severe atopic dermatitis but included also subjects with comorbid asthma (51) confirmed the efficacy of dupilumab in pediatric asthma. The review proved a significant reduction of AERs under dupilumab, especially in T2-high patients, alongside improvements in pre-bronchodilator FEV₁/forced vital capacity (FVC) z-scores after 52 weeks. Safety was favorable, and although FeNO, IgE, and eosinophil counts did not significantly change, quality of life improved meaningfully (50). Consistent findings emerged from a retrospective real-life Chinese study, which reported reduced exacerbation rates and improved FEV₁ in children (6–14 years) with moderate-to-severe T2-high asthma after one year of treatment with dupilumab (52).

Indirect comparisons between dupilumab and other biologics, mostly derived from systematic reviews and network meta-analyses, seem to suggest that dupilumab may provide greater exacerbation reduction and FEV₁ improvements compared with mepolizumab and omalizumab, particularly in T2-high asthma regardless of allergic status (53). However, these trials included mostly adults. Similarly, the real-world US ADVANTAGE study (dupilumab *n* = 2,138 vs. omalizumab *n* = 1,313) indicate a possible advantage of dupilumab in terms of asthma attacks and OCS-sparing effects compared to omalizumab, although the adolescents included were few (54). Finally, in network meta-analyses of 2,672 pediatric subjects, dupilumab seemed to have the highest efficacy in terms of exacerbation prevention, whereas omalizumab maintained the most favorable safety profile (55).

Collectively, available studies seem to support the use of dupilumab in school-aged children and adolescents with T2-high severe asthma to reduce exacerbations and improve lung function. However, conclusions on its relative superiority over other biologics in pediatric asthma rely predominantly on adult data and limited pediatric subgroup analyses. Moreover, due to the current absence of specific head-to-head pediatric trials, caution is required in interpreting the comparative efficacy of pediatric biologic treatments. Indirect cross-trial comparisons inevitably limit the strength of inferences, so conclusions must be considered hypothesis-generating rather than definitive.

Worth mentioning, the ongoing phase 3 **LIBERTY ASTHMA TREKIDS** study is investigating long-term efficacy and safety of dupilumab in preschool children (2–5 years), paving the way for treating preschooler wheezing with biologics (56).

As regards safety, dupilumab is generally well tolerated in pediatrics. Most common adverse events are mild injection-site reactions, conjunctivitis, rhinopharyngitis, and transient hypereosinophilia (>3,000 cells/mL), which is self-limited and rarely requires discontinuation (57, 58). As with other biologics, parasitic infections and rare anaphylaxis have been reported, therefore supervised hospital administration is recommended for initial dosing.

## 5 Mepolizumab

Mepolizumab is a humanized monoclonal IgG1 antibody targeting IL-5, an interleukin involved in eosinophil maturation, survival and activation (59). In Europe, mepolizumab is licensed for children ≥6 years with refractory severe eosinophilic asthma (blood eosinophils ≥300 cells/µL or ≥150 cells/µL plus regular OCS use) (60). Mepolizumab is administered subcutaneously every 4 weeks, with an age-dependent dose of 40 mg for children aged 6–11 years or 100 mg for subjects ≥12 years. Whereas the National Institute for Health and Care Excellence (NICE) guidelines suggest to stop treatment in the absence of a 50% reduction of asthma attacks or OCS-dose after one year of treatment (61), GINA documents recommend a first reassessment after 4 months of therapy (4). Currently, mepolizumab is also approved as an add-on therapy for eosinophilic granulomatosis with polyangiitis in children ≥6 years. The pediatric use in chronic rhinosinusitis with nasal polyposis and uncontrolled hypereosinophilic syndrome remains investigational despite limited evidence of efficacy and ongoing age-specific trials (62).

In the field of severe asthma, mepolizumab age-specific evidence is limited, since phase 2 and phase 3 RCTs included only 37 adolescents among a total of 1,878 participants (63). Evidence for children younger than 12 years is even more limited and largely derived from safety and pharmacokinetic studies rather than clinical efficacy trials. The main RCTs on mepolizumab including pediatric participants were the Dose Ranging, Efficacy, And safety with Mepolizumab in severe asthma (**DREAM)** (64); the MEpolizumab treatment iN patients with Severe eosinophilic Asthma (**MENSA)** (65); the SteroId ReductIon with mepolizUmab Study (**SIRIUS)** (66); and the Mepolizumab adjUnctive therapy in subjects with Severe eosinophiliC Asthma (**MUSCA)** (67). Notably, the Mechanisms Underlying asthma Exacerbations Prevented and Persistent With Immune-Based Therapy: A Systems Approach – Phase 2 (**MUPPITS-2)** was the only fully pediatric trial in this field (68) (Table 3).

**Table 3:** Characteristics and results of main studies on mepolizumab involving pediatric subjects.

Trial	Population	Pediatric subjects/total	Dosing	Treatment duration (weeks)	Efficacy in Pediatrics
DREAM	Adolescents (≥12 y) + adults	9/621	IV 75, 250 or 750 mg Q4W	52	Dose-dependent significantly reduced asthma attacks (no pediatric-specific results); Improvements of lung function, ACQ-6, AQLQ (n.s.)
MENSA	Adolescents (≥12 y) + adults	25/576	IV 75 mg or SC 100 mg Q4W	32	Significantly reduced asthma attacks; Significantly increased FEV₁; Significant improvement of asthma control and QoL (no pediatric-specific results)
SIRIUS	Adolescents (≥12 y) + adults	2/135	SC 100 mg Q4W	24	Significantly reduced OCS dose and Eo; Significant improvement in SGRQ and ACQ-5; No FEV₁ improvements (no pediatric-specific results)
MUSCA	Adolescents (≥12 y) + adults	9/551	SC 100 mg Q4W	24	Significantly reduced asthma attacks; Significant improvement in SGRQ, ACQ-5 and FEV₁
MUPPITS-2	6–17 y	290/290	SC: 40 mg (<40 kg) or 100 mg (≥40 kg) Q4W	52	Significantly reduced asthma attacks (especially during fall season) and Eo; No improvement of lung function or CASI

ACQ, Asthma Control Questionnaire; AQLQ, Asthma Quality of Life Questionnaire; CASI, Composite Asthma Severity Index; Eo, eosinophils; FEV₁, forced expiratory volume in 1 s; IV, intravenous; n.s., not significant; OCS, oral corticosteroids; Q4W, every 4 weeks; QoL, quality of life; SC, subcutaneous; y, years; SGRQ, St George's Respiratory Questionnaire.

The DREAM study (*n* = 621, 9 adolescents) was a phase IIb/III RCT on severe eosinophilic asthma (eosinophils ≥150/µL at screening or ≥300 in the previous year) evaluating intravenous mepolizumab. The study demonstrated a dose-dependent reduction in asthma exacerbations together with reduced sputum eosinophils over 52 weeks, but no significant improvements in terms of lung function, asthma control, QoL, or OCS use (64).

The MENSA trial (*n* = 576, 25 adolescents) was a 32-week phase III RCT comparing intravenous and subcutaneous mepolizumab. The trial showed a significant reduction in exacerbations with both regimens, paired with improved asthma control and quality of life, although pre-bronchodilator FEV₁ gains were modest (about 100 mL) (65). Further systematic reviews proved mepolizumab effects on lung function to be scarcely consistent among adults (69), with even greater variability in FEV₁ response in the case of children and adolescents (63). Nevertheless, an adult study proved that patients under mepolizumab may undergo both early (within 1 month) and late (after 6 months) response in terms of FEV₁ improvement, with early response being much likely in the presence of lower baseline FEV₁ and higher blood eosinophils count (70). Another study proved that most benefit in terms of FEV₁ were observed in patients with higher baseline sputum eosinophils, suggesting that airway inflammation may predict mepolizumab response better than eosinophilia (71).

The SIRIUS trial, a 24-week phase III study in adults with steroid-dependent eosinophilic asthma, demonstrated a significant OCS-sparing effect with improved asthma control, quality of life, and exacerbation rates, but no FEV₁ improvement. Pediatric conclusions were limited by minimal adolescent inclusion (66). Similarly, the phase IIIb MUSCA trial (*n* = 551, 9 adolescents) confirmed reductions in asthma exacerbations, with modest improvements in terms of asthma control, quality of life and pre-bronchodilator FEV₁ (+110 mL) (67).

MUPPITS-2, the only fully pediatric phase III RCT, enrolled 290 subjects aged 6–17 years with exacerbation-prone eosinophilic asthma, mostly of African American origins. In those subjects, mepolizumab modestly reduced exacerbations over 52 weeks (particularly during the fall season) while lowering serum and nasal eosinophils. The more sustained reduction of asthma attacks during fall season suggests a possible role in viral-triggered asthma attack prevention (8), similarly to omalizumab. However, the overall reduction in asthma attacks was less marked compared to previous studies (72), and no improvements in lung function, asthma severity, FeNO, or time to first exacerbation (68) were detected. These findings may reflect distinct pediatric asthma biology, including lower IL-5 expression in BAL (58) and weaker correlations between blood and airway eosinophils, as well as potential ethnicity-related effects. In this regard, nasal transcriptomic analyses suggested greater benefit in children with eosinophilic inflammatory signatures, while mixed endotypes seem to be associated with poorer response (44, 68).

Post-hoc meta-analyses suggest comparable exacerbation reduction in both adolescents and adults undergoing mepolizumab (63), despite wide confidence intervals due to small sample size. As regards younger children, an open-label study of 36 children aged 6–11 years treated with subcutaneous mepolizumab showed a substantial reduction of asthma attacks with sustained safety over one year, although without lung function improvement (73). The study was followed by a one-year open-label extension, which confirmed long-term safety and efficacy up to two years (72). Finally, a Cochrane review of 17 RCTs including about 7,600 participants treated with anti-IL-5 and IL-5R biologics (namely, mepolizumab, reslizumab, and benralizumab) confirmed a significant 50% reduction in exacerbations within 16 weeks of mepolizumab treatment, without major safety concerns (74). On the contrary, improvements in lung function and quality of life remained modest and inconsistent, particularly in pediatric populations. Real-life studies seem to confirm less pediatric efficacy of mepolizumab (*n* = 16) compared to omalizumab (*n* = 60) (75).

To address the scarcity of pediatric evidence on mepolizumab, the Treating Severe Pediatric Asthma (TREAT) trial is an ongoing randomized controlled non-inferiority trial that is currently being performed to provide a head-to-head comparison between mepolizumab and omalizumab in terms of asthma attacks in subjects aged 6–17 years during 52 weeks of treatment (76). This will be the first head-to-head trial in pediatric severe asthma and will hopefully provide relevant information to help clinicians in choosing the best biologic for their patients.

The long-term safety profile of mepolizumab was good in all pediatric studies similarly to adulthood, with headache as the commonest side effect, followed by local injection site reaction, backache and upper respiratory infections. Contrarywise, herpes simplex re-activations seem less likely in children compared to adults (44). Notably, serious adverse events – mostly respiratory and infectious events – were apparently more frequent in pediatrics (3/15 children 6–11 years; 2/9 adolescents 12–17 years; 34/502 adults), mostly due to the small size of the pediatric sample. As for other biologics, parasitic infections must be ruled out and treated before starting mepolizumab. Anaphylaxis occurs in less than 1% of the subjects, and requires the same recommendation as other biologics (60). As regards immunogenicity, phase III RCTs detected anti-drug antibodies (mostly non-neutralizing) in 4% of adult patients under mepolizumab treatment (66). To our knowledge, there is a lack of pediatric data on the field.

Interestingly, RCTs evaluating treatment discontinuation demonstrated that adult patients who discontinued mepolizumab experienced increased asthma attacks approximately 16 weeks post-cessation, with blood eosinophil levels and asthma attack frequency tending to return to pre-treatment baseline within 3–6 months (77, 78).

## 6 Tezepelumab

Tezepelumab is the most recently approved biologic in the field of pediatric asthma. It is a human IgG2 monoclonal antibody targeting the thymic stromal lymphopoietin (TSLP), an epithelial-derived alarmin released in response to allergens, viruses, and pollutants. Interestingly, higher airway TSLP levels seem to correlate with increased asthma severity (79). TSLP activates dendritic cells, ILCs and T and B lymphocytes (80), orchestrating both T2-high and T2-low asthma pathways, including TH17 polarization and IL-17-mediated neutrophilic inflammation (81). Accordingly, Tezepelumab is the first biologic to be effective across all asthma endotypes (82), providing broad inhibition of both upstream T2-high and T2-low responses.

In Europe, tezepelumab is currently authorized for children ≥12 years with severe uncontrolled asthma, regardless of the endotype. It is administered subcutaneously at the dose of 210 mg monthly. NICE recommends reassessment after 12 months, with discontinuation or switch to another biologic if asthma attacks are not halved (83).

Key clinical pediatric evidence comes from the **PATHWAY** (84) and **NAVIGATOR** (85) trials, as well as the **DESTINATION** extension study (86) (Table 4), all including adolescents≥12 years. To our knowledge, no RCTs addressing tezepelumab efficacy and safety in children 6–11 years are currently available, but phase I studies in children from 5 to 11 years old showed promising pharmacokinetics, pharmacodynamics and safety profile with monthly subcutaneous administration of 70 mg (87). Phase III trials are underway (88).

**Table 4:** Characteristics and results of main phase-3 studies on tezepelumab involving pediatric subjects.

Trial	Population	Pediatric subjects/total	Dosing	Treatment duration (weeks)	Efficacy in Pediatrics
NAVIGATOR	Adolescents (≥12 y) + adults	82/1,061	SC 210 mg Q4W	52	Reduced AER in adolescents (n.s.); Increased FEV₁ in adolescents (n.s.)
PATHWAY	Adolescents (≥12 y) + adults	30/550	SC 210 mg Q4W	52	Significant reduction of asthma attacks (no pediatric-specific results);
DESTINATION	Adolescents (≥12 y) + adults	50/918	SC 210 mg Q4W	104	Long-term asthma attacks reduction (no pediatric-specific results)

AER, asthma exacerbation rate; FEV₁, forced expiratory volume in 1 s; n.s., not significant; Q4W, every 4 weeks; SC, subcutaneous; y, years.

The phase 3 PATHWAY trial (*n* = 550, 30 adolescents) demonstrated a significant reduction in asthma exacerbations over 52 weeks of tezepelumab treatment, although results were not stratified by age (84). By contrast, the phase 3 NAVIGATOR study (*n* = 1,061, 82 adolescents) provided results stratification both by eosinophilia and by age. In adults, tezepelumab significantly reduced AERs and improved pre-bronchodilator FEV₁, regardless of FeNO, IgE, blood eosinophils or allergic status (85). Adolescents showed similar trends in FEV₁ improvement and asthma attack reduction, although statistical significance was limited by small sample size, highlighting the need for age-specific studies (85). NAVIGATOR *post-hoc* analyses proved consistent efficacy across gender, body mass index (BMI), asthma endotype and allergic status, with a notable 64% reduction in asthma exacerbations among dupilumab-eligible patients (89). Exacerbations were reduced along all seasons, suggesting effectiveness against both viral- and allergen-induced asthma attacks (90). Further *post-hoc* analyses indicated rapid (within 2 weeks) and sustained (over 52 weeks) significant improvements in FEV₁, FVC and PEF (peak expiratory flow), regardless of ICS therapy. Additional improvements in FEV₁/FVC and FEF25–75 (forced expiratory flow between 25% and 75% of FVC) were also detected, although not significant (91). Improvements were greater in patients with higher reversibility (FEV₁ post ≥20%), shorter disease duration (<20 years), and lower baseline FEV₁/FVC (<0.7), suggesting benefit from early intervention even in the case of severe obstruction (91). Since all participants had documented FEV₁ reversibility, extrapolation cannot be made to subjects with fixed obstruction. Finally, the DESTINATION extension study confirmed long-term safety and sustained asthma attack reduction up to two years of treatment, though pediatric-specific conclusions remain limited. Interestingly, after two years of treatment, patients were followed-up until 40 weeks post-discontinuation, showing a progressive partial reversion of biomarkers, clinical outcomes and functional parameters. These results suggest that long-term treatment is required to maintain full therapeutic benefit, at least in adults (86).

As regards biologic comparisons, a recent systematic review applying Bayesian meta-analysis indirectly compared tezepelumab with dupilumab, benralizumab and mepolizumab in patients ≥12 years with eosinophilic asthma (≥300/µL). Among these biologics, tezepelumab was associated with more favorable asthma exacerbations reduction, whereas dupilumab seems to correlate with better FEV_1_ improvements, although lung function differences were below clinically meaningful thresholds (92). However, these comparative results rely on indirect cross-trial comparisons, so conclusions cannot be readily generalized, even to adults. Pediatric-specific head-to-head studies are still needed to confirm whether these comparative differences apply to children, too.

## 7 Standardizing biologic response and asthma remission definitions in pediatric severe asthma

At present, one of the greatest difficulties in studies involving severe asthma is the lack of standardized, univocal definitions of both response to biologics and asthma remission. Accordingly, a 2023 systematic review applying COSMIN (COnsensus-based Standards for the selection of health Measurement INstruments) methodology identified 96 candidate and 24 priority outcome measures for severe asthma (93). Among them, only the children asthma control test (C-ACT) demonstrated adequate validity and responsiveness in children. These findings highlighted the need for new validated, severe asthma-specific, pediatric-specific and patient-centered measures. To address these gaps, a multistakeholder consensus established the pediatric Core Outcome Measure set for Severe Asthma (COMSA), comprising FEV₁ z-scores, annual severe exacerbation rate, maintenance OCS use, PAQLQ, and C-ACT or ACT according to age, to standardize biologic trials and enable cross-study comparisons (94).

**Figure 1:**
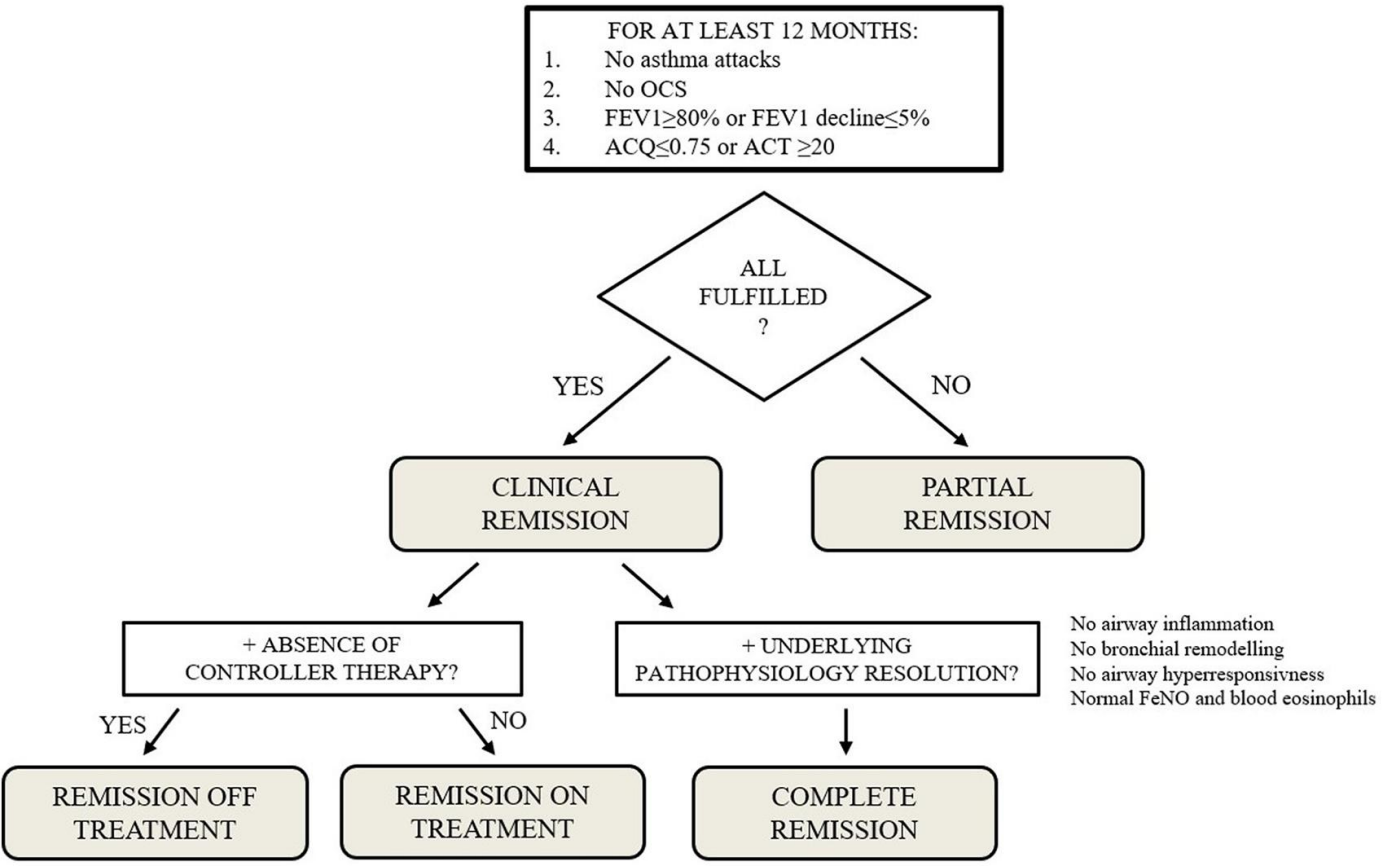
Proposed algorithm to define asthma remission, according to the definitions provided by Menzies-Gow et al, 2020 (91). ACQ, Asthma Control Questionnaire; ACT, asthma control test; FeNO, Fractional exhaled Nitric Oxide; FEV_1_, forced expiratory volume at 1 s; OCS, oral corticosteroids.

In parallel, new definitions and scores were proposed, to standardize biologic response and the assessment of asthma remission. In that regard, a 2022 Delphi consensus proposed four clinical and functional remission states, combining clinical control with pathophysiological resolution: partial remission; clinical remission *on* treatment; clinical remission *off* treatment; and complete remission (95). Once reached clinical remission, the authors suggest that anti-asthmatic treatments should be stepped down (“remission *on* treatment”) virtually until the absence of any ongoing controller therapy (“remission *off* treatment”), at least for mild asthma cases. Figure 1 proposed an algorithm to evaluate asthma remission according to the definitions provided in the 2022 Delphi consensus.

Interestingly, *post-hoc* analyses of QUEST and TRAVERSE study applying these definitions of clinical remission showed that dupilumab induced clinical remission in about 37% patients after one year of treatment and in about 27% patients after two years (96). Further pediatric studies are needed to better define the potential of each biologic in achieving clinical remission.

Even more intriguingly, another consensus of international healthcare experts and severe asthma patients attempted to provide objective, patient-centered tools to quantify response to biological therapies for severe asthma. Those efforts lead to the development of a pediatric-specific and adult-specific CompOsite iNdexes score For Response in asthMa (CONFiRM) score, integrating COMSA-derived outcomes (97). CONFiRM score ranges from −31 (“deterioration”, which means worsening of asthma after starting biologic treatment) to 69 (best possible response to biologic, defining “super-responders”) (97). If further studies will validate the pediatric CONFiRM score, this could be a unique tool to quickly and objectively assess and compare biologic response.

## 8 Predictors of good and poor biologics response in pediatric severe asthma: state of the art

Approximately 75% of patients with severe asthma meet overlapping eligibility criteria for two or more biologics (98). Therefore, the identification of predictive biomarkers of good response and criteria for timely therapeutic switching represents a critical area of research. In this context, there is growing interest in age-specific biomarkers capable of predicting biologic response in children, with the aim of improving pediatric cost-effectiveness. As a matter of fact, non-responder rates among adolescents with severe asthma treated with biologics have been reported to be as high as 60% (99).

When interpreting biomarkers to guide biologic selection, it is essential to distinguish regulatory eligibility criteria from biomarkers associated with clinical response, as these concepts do not always overlap. Regulatory thresholds identify patients eligible for treatment, but do not necessarily predict therapeutic efficacy. This distinction is particularly relevant for omalizumab, whose eligibility is based on serum IgE levels and allergen sensitization, whereas clinical response is more closely associated with markers of type-2 inflammation such as blood eosinophils and FeNO. In pediatric asthma, biomarker interpretation is further complicated by age-related physiological variability, the lack of pediatric-validated cut-offs across age groups, and the influence of infections, atopy, comorbidities, treatment adherence, and growth, which renders some adult asthma biomarkers, such as serum periostin, unreliable in children (100).

Currently, sputum eosinophils, blood eosinophils and FeNO are the most commonly used biomarkers in T2-high asthma, whereas T2-low asthma biomarkers are exploratory. Severe eosinophilic asthma with both higher risk of asthma attack and maximal possibility to respond to T2-high-targeting biologics is commonly identified by the presence of sputum eosinophils>2% (101), blood eosinophils>150*μ*L (102) and FeNO levels >20 ppb (103). although these thresholds should be interpreted with caution in pediatric populations. Interestingly, total serum IgE has been shown to correlate with asthma severity in both adults and children (104), but does not reliably predict response to biologic therapy. As regards T2-low asthma, sputum neutrophils >40%, IL-17, IL-8, myeloperoxidase (MPO) and neutrophil elastase have been proposed (105, 106), but require further pediatric validation. It is important to remember that, in pediatrics, blood eosinophil counts are higher and more variable than in adults, making adult-derived thresholds only approximate surrogates of eosinophilic disease. Contrarywise, sputum eosinophils are more specific but rarely feasible in routine pediatric practice (107). FeNO is a useful non-invasive marker of IL-13–driven inflammation and good predictor of response to anti-IL-4R*α* therapy, but its values are influenced by many factors like age, rhinitis, smoke and inhaled corticosteroid adherence (107). Consequently, real-life pediatric data and longitudinal follow-up remain essential to contextualize biomarker values and support individualized treatment decisions in pediatric severe asthma.

**Figure 2:**
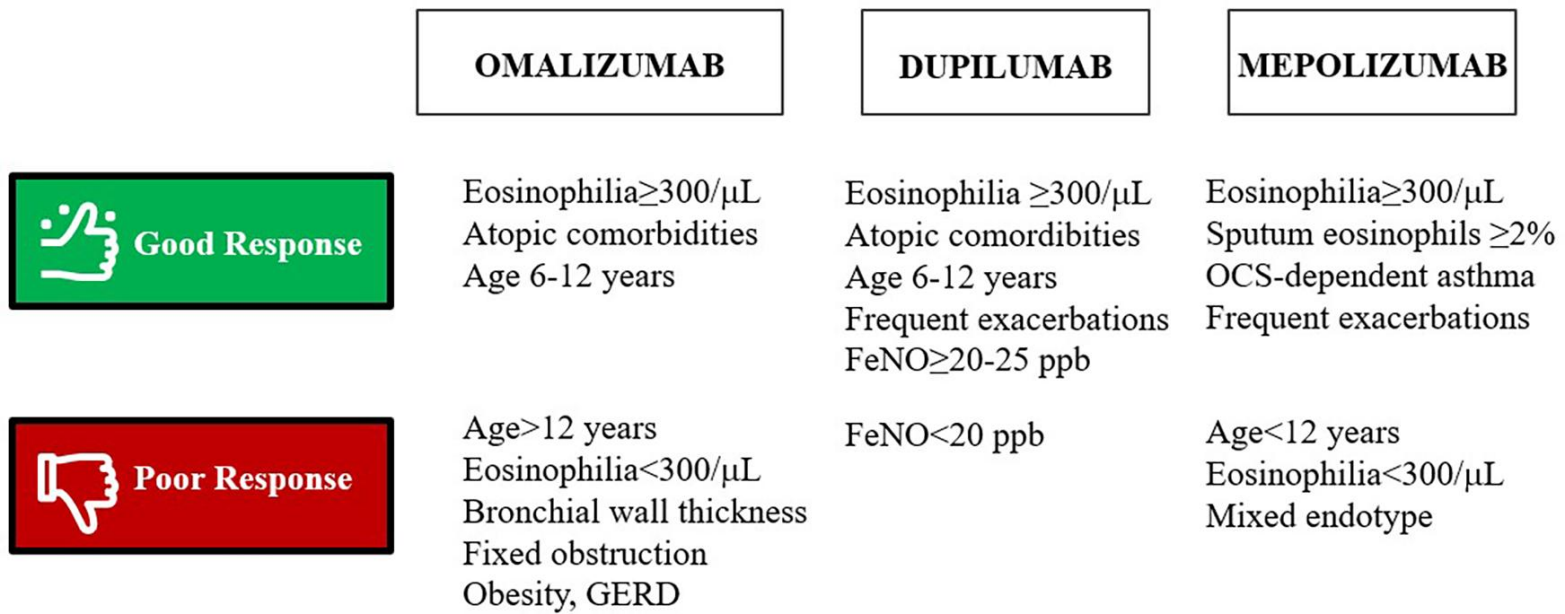
Main pediatric-specific predictors of good and poor response to biologics in severe asthma according to the literature. FeNO, Fractional exhaled Nitric Oxide; GERD, gastroesophageal reflux; OCS, oral corticosteroids; ppb, parts per billion.

Looking at currently available biomarkers and literature findings, a proposal of good and poor biologic response predictors in pediatrics is provided in Figure 2.

In meta-analysis, omalizumab response appears to correlate with eosinophilia, while baseline total IgE does not reliably predict treatment response beyond defining eligibility and dosing range. By contrast, higher baseline FeNO seems to identify patients more likely to benefit from omalizumab treatment. (108) Therefore, although total IgE decreases during omalizumab treatment, basal IgE levels should not be considered a reliable predictor of clinical response (58). Dupilumab seems to benefit mostly adolescents with high baseline FeNO, independently from eosinophilia or other clinical factors (109). This is in line with the evidence that IL-4/IL-13 directly increase nitric oxide (NO) production, therefore making FeNO a good biomarker for dupilumab response (58). Another study proved that both FeNO and blood eosinophils are good predictors of lung function response in adolescents undergoing dupilumab treatment (45). Finally, studies proved better mepolizumab response in the presence of higher sputum eosinophils or frequent prior exacerbations (110), although pediatric-specific conclusions remain limited. Contrarywise, FeNO seemed not a good predictor of mepolizumab response (111).

As regards predictors of poor response, a small pediatric case-series by Tsuge et al. reported that increased bronchial wall thickness on chest CT, an indirect marker of airway remodelling, may relate to poor clinical and functional response to omalizumab, therefore paving the way for radiologic predictors of poor response to biologics (112). Age >12 years, asthma exacerbations or hospital admission in the previous 6 months and comorbidities such as obesity or gastroesophageal reflux are suggested as poor response criteria to omalizumab whereas FeNO<20 ppb and blood eosinophils <300cells/*μ*L seems to suggest poor response to dupilumab and mepolizumab, respectively (113).

In the future, the definition of pediatric-specific biomarkers from exhaled breath as well as microbiome and “omics” approaches (metabolomics, epigenomic or transcriptomics signatures) may be beneficial, with microRNA signatures and lung microbiome emerging as promising predictors of biologic response in severe pediatric asthma (82, 114, 115).

## 9 Future directions in biologic treatments for pediatric severe asthma

Currently, several emerging research directions are addressing new indications, dosing strategies and delivery methods for anti-asthmatic biologics.

One of the most intriguing perspectives is the disease-modifying potential of early biologic treatments, which may have a preventive role in preschool wheezing, seasonal exacerbations and even in asthma onset. The ongoing multicenter *Preventing Asthma in High-Risk Kids* (PARK) RCT is enrolling about 250 children aged 24–47 months with recurrent wheezing, early aeroallergen sensitization and asthmatic or atopic relatives, to undergo two years of monthly omalizumab administration followed by two years of observation (116). Outcomes will include allergic sensitization, lung function, atopic comorbidities, Composite Asthma Severity Index (CASI) score, symptoms, healthcare use, and safety. If effective, omalizumab could offer a cost-efficient preventive alternative to environmental control, with the added benefit of reducing viral respiratory susceptibility. In that regard, the *Preventive Omalizumab or Step-up Therapy for Severe Fall Exacerbations* (PROSE) study tested a 4-month seasonal short course of omalizumab to be started 4–6 weeks before school return in 513 inner-city asthmatic children and adolescents (117). By restoring interferon-α responses to rhinovirus, omalizumab significantly reduced fall exacerbations (118). Those results are consistent with other studies proving a decreased incidence, duration, and viral shedding of rhinovirus infections in allergic asthmatic children undergoing omalizumab (119).

Biologic applicability beyond the currently licensed cut-offs is another hot topic in pediatric severe asthma. Current eosinophils and IgE thresholds are adult-derived and may not be appropriate for younger children, who naturally exhibit higher and more variable values depending on age, sex, and BMI (58, 59). Consequently, up to one-third of children who could benefit from omalizumab fall outside its licensed dosing criteria (23). Recent studies proved complete omalizumab efficacy even in children with IgE>2000 kU/L (120, 121), with significantly reduced exacerbations, healthcare utilization and OCS paired with improved lung function (29, 121).

Flexible and tailored dosing strategies are also critical: while most biologics are dosed according to age, omalizumab dose is weight-dependent, which may influence its efficacy compared to other age-based biologics (58). In this regard, pharmacokinetic modeling of dupilumab showed that, after weight correction, drug exposure is similar across all ages, supporting a weight-adjusted dosing instead of the age-dependent one (122). Therefore, pediatric trials are essential to refine dosing strategies and optimize responses across diverse pediatric populations.

**Figure 3:**
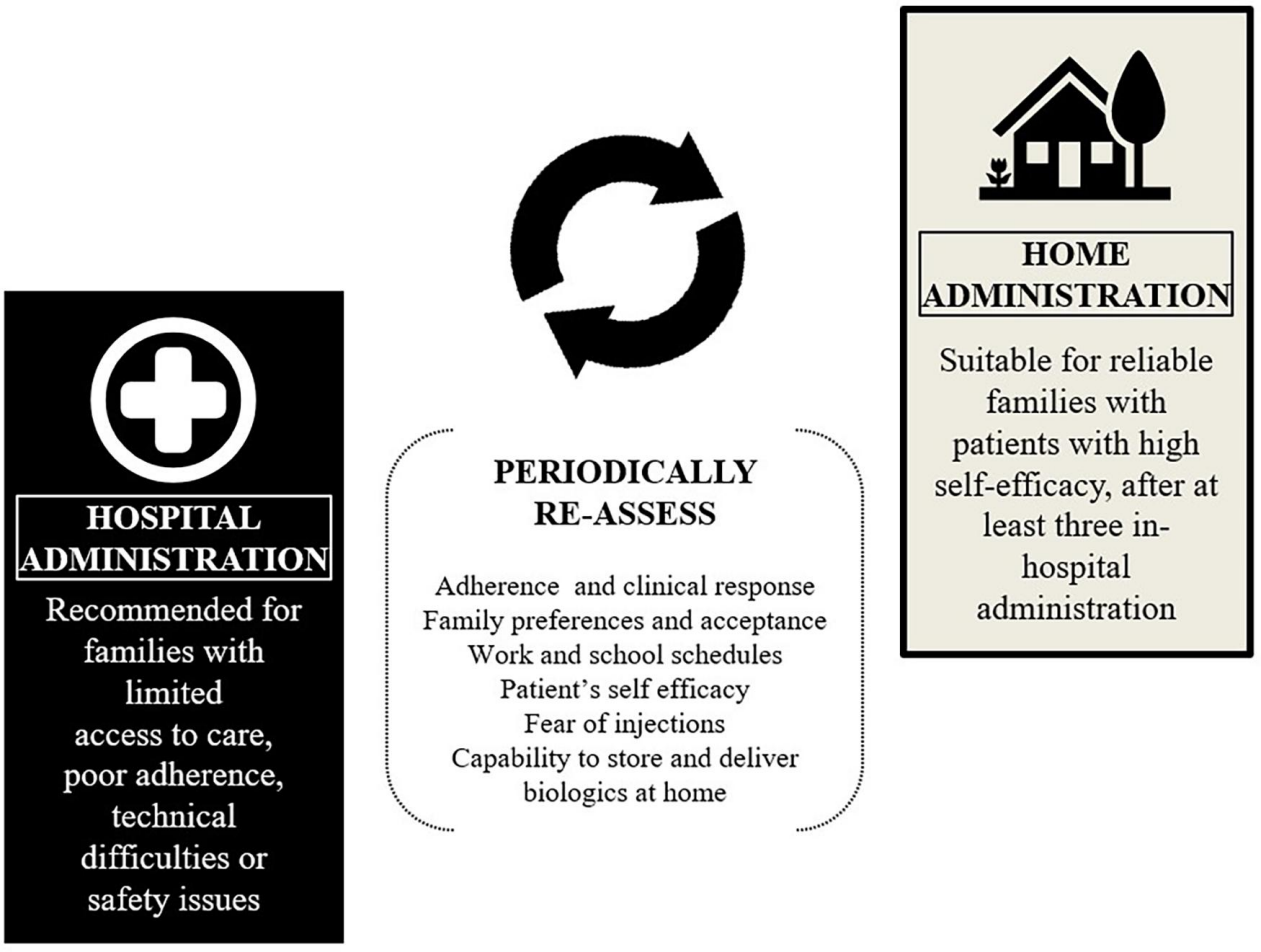
Factors to assess in considering shift from hospital to home-delivery of biologics in pediatric asthma.

New ways to deliver biologics are also under investigation, with the goal of increasing convenience, adherence, and accessibility. These include home administration, inhaler formulations and ultra-long-acting molecules enabling extended dosing intervals. Especially after the 2020 pandemics, the opportunity of biologic home administration has gained particular attention as a cost-effective, family-friendly alternative to hospital-based injections. In the PATH-HOME study (*n* = 216, 24 adolescents) self-administrations of tezepelumab using prefilled syringes and autoinjectors was both safe and effective (123). While barriers remain - such as fear of needles, technical challenges and need of caregivers’ and patients’ specific training - pediatric studies suggest that home-based administration can improve both asthma control and quality of life, with high levels of family acceptance (124). Further pediatric-specific studies are required, to assess acceptability, feasibility and patient-centered perceptions of home administration of biologics. Due to safety reasons, at least three hospital administrations are advisable before considering home use (35). Factors such as adherence, clinical response, family preferences, technical difficulties and patients self-efficacy should be re-assessed periodically (16) (Figure 3).

Inhaled biologics, such as ecleralimab (anti-TSLP) are currently under phase II trials (125), in the attempt to offer targeted lung delivery with fewer systemic effects. Moreover, ultralong-acting biologics to be administered twice-yearly, like depemokimab (subcutaneous anti-IL-5), could reduce injection burden while improving treatment adherence (126). While adult and adolescent data are promising, pediatric evidence - particularly in children under 12 - is still needed.

Finally, combination therapies are emerging for patients with overlapping inflammatory pathways or comorbidities. Although not yet guideline-recommended, case reports and real-world data suggest that concomitant biologic treatments could be safe and effective if proven cost-effective (127). For example, a 12-year-old with severe asthma was successfully managed with six months of combined omalizumab plus mepolizumab treatment, without adverse effects (128). Similarly, combining biologics with allergen immunotherapy (AIT) - the only current disease-modifying therapy for IgE-mediated allergy - may enhance outcomes (129). Pediatric studies, particularly with dust mite AIT plus omalizumab, indicate improved asthma control, lung function, and FeNO without safety concerns, supporting a tailored approach to be evaluated on a case-by-case basis, especially in non-responders (130–132).

## 10 Conclusions

Severe pediatric asthma is a rare yet burdensome disease. Over the last two decades, biologic therapies have substantially expanded and deeply changed the treatment of severe asthma, leading to meaningful clinical and functional efficacy with a favorable safety profile. Unfortunately, most studies and clinical trials on the field were primarily conducted in have focused on adults, leaving a relevant gap in pediatric-specific evidence, which is still limited in quality.

**Figure 4:**
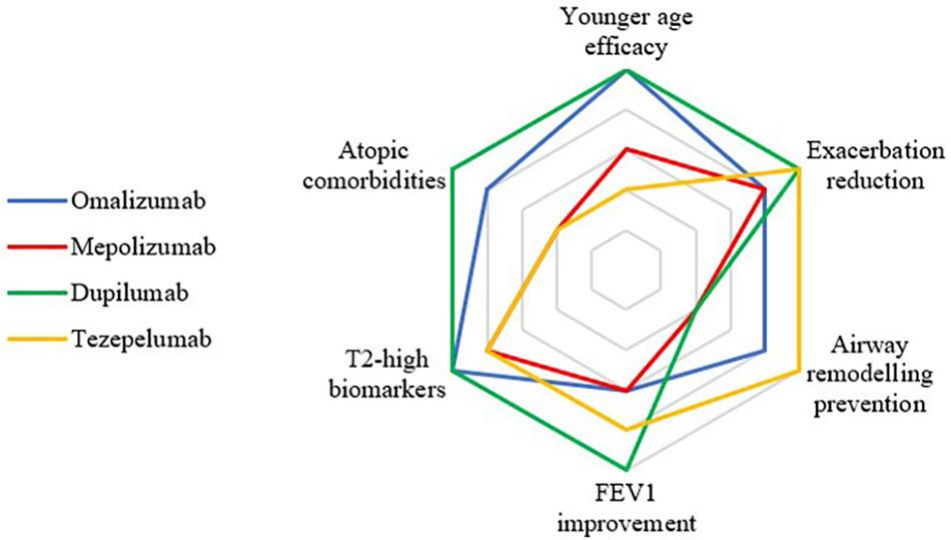
Qualitative evidence map of perceived strengths of currently EU-licensed biologics in pediatric severe asthma across age, comorbidities, biomarkers, and clinical and functional outcomes. Each domain is shown on an illustrative 1–5 scale that reflects the apparent strength and consistency of pediatric-relevant evidence within that domain, based on indirect, cross-trial comparisons and real-world data, and is meant as a descriptive, hypothesis-generating summary rather than a definitive ranking of comparative efficacy. Omalizumab is supported by extensive pediatric experience and consistent data on good exacerbation reduction, especially in younger children with T2-high asthma, and may have a role in airway remodelling prevention. Dupilumab has robust pediatric RCT evidence for reduction in exacerbations and shows particularly strong signals for lung-function improvement, especially in younger children with atopic comorbidities. Tezepelumab demonstrates broad efficacy in reducing exacerbations in adolescents with both T2-high and T2-low asthma, with emerging data suggesting potential effects on remodelling. Mepolizumab reduces exacerbations in eosinophilic pediatric asthma, while lung-function benefits appear modest and more variable, in the context of a smaller pediatric evidence base. Importantly, the choice of biologic for an individual child must be made case-by-case after multidimensional evaluation and cannot rely solely on this schematic representation.

In the last years, the landscape of pediatric asthma biologic research has expanded through a combination of fully pediatric trials, adolescent subgroups within adult RCTs, post-hoc analyses and real-world cohorts, supporting the efficacy and safety of dupilumab, mepolizumab, omalizumab, and tezepelumab in children and/or adolescents. Emerging data from pediatric-specific evidence suggest that different biologics may have slightly different efficacy profiles across outcomes and subgroups (Figure 4). However, these hypotheses are largely based on indirect comparisons, and remain to be confirmed by pediatric-specific head-to-head trials. Therefore, any apparent differences among biologics across outcomes or subgroups should be regarded as hypothesis-generating signals rather than definitive efficacy rankings. Importantly, the selection of one molecule over another does not follow an absolute rule and should instead be individually tailored according to age, relevant biomarkers, comorbid conditions and other multidimensional issues.

Omalizumab, as the longest-standing biologic in pediatric severe asthma, is supported by the largest body of pediatric data, including RCTs and long-term real-world studies in school-age children and adolescents. It is indicated for severe allergic asthma and is particularly effective in preventing allergen-induced and virus-induced exacerbations. It appears most beneficial in children with atopic comorbidities, good bronchodilator response and higher baseline FeNO. On the contrary, baseline total IgE level, although an eligibility criterion, seems not to reliably predict treatment response, and current evidence does not support its use as a stand-alone treatment-response or remission target. Improvements in lung function seem to be modest, especially in the presence of fixed obstruction, but its potential role in preventing airway remodeling and seasonal exacerbations before school return is under investigation. Benefits seem to be sustained for up to two years and may persist after discontinuation.

Dupilumab has the most robust fully pediatric RCT dataset among current biologics. It addresses T2-high asthma, leading to lower exacerbation rates and appears effective in reducing asthma exacerbations in pediatric populations. Among the currently available pediatric studies, indirect comparisons and network meta-analyses suggest that dupilumab may provide the greatest and earliest improvements in lung function, especially in the presence of high FeNO and high eosinophils regardless of allergy. However, due to the quality of the currently available studies, the magnitude of this effect should be considered hypothesis-generating and will require more high-quality pediatric studies to be confirmed. Efficacy is high in the presence of atopic comorbidities, and adolescent data from QUEST, TRAVERSE and combined analyses, together with real-world cohorts, seem to support sustained efficacy and favorable safety over several years after withdrawal.

Mepolizumab has limited pediatric evidence, especially in children aged 6–11 years, due to the presence of only one fully pediatric RCT (MUPPITS-2) and small open-label studies. Across these studies, improvements in lung function have been modest and inconsistent. Available data suggest that mepolizumab may be particularly useful in reducing exacerbations in eosinophilic asthma, particularly in the presence of high blood or sputum eosinophils, high IL-5 activity, and frequent OCS use. Since IL-5 sputum concentrations increase with age, it is expected to suit older children better and may prevent viral-induced exacerbations. Current studies suggest that a partial loss of efficacy can occur within 16 weeks of withdrawal, but the extent and durability of benefit remain not fully defined in children.

Finally, tezepelumab is the only biologic currently suitable for all asthma endotypes, with proven efficacy in reducing exacerbations and improving lung function in children >12 years in both phase 3 trials and post-hoc analyses. It also seems to reduce FeNO and may show promise in non-responders to other biologics or in subjects with baseline severe obstruction, although lung function gains seem greater in patients with preserved bronchodilator reversibility. Current evidence supports continuation beyond two years for sustained benefits. However, pediatric evidence is limited and currently restricted to adolescents included in adult-dominated trials, with a complete absence of efficacy trials in younger children and of head-to-head pediatric comparisons. Therefore, dedicated trials in children <12 years are urgently needed for age-specific efficacy data.

Importantly, these therapeutic considerations must be interpreted in light of both the strengths and limitations of this review. The narrative adopts a pediatric-centered perspective, integrating evidence from RCTs, real-world studies, and recent consensus initiatives, with a focus on age-specific predictors of good and poor response, standardized outcomes, and future innovations guiding precision biologic selection. However, the narrative nature of the review, the heterogeneity of endpoints and study designs - mostly featuring small pediatric cohorts and applying adult-derived biomarker cut-offs - and the lack of head-to-head trials limit the strength of inference, particularly for children under 12 years of age or for newer agents like tezepelumab. Moreover, no conclusions can be drawn for preschool wheezers, where biologics remain off-label at present.

In the future, dedicated multicenter trials, real- world cohorts, and rigorous randomized head-to-head comparisons are needed across preschool, school-age, and adolescent populations of diverse ethnic backgrounds, to close the evidence gap in pediatric severe asthma. Pediatricians could play a central role in generating robust, pediatric-specific evidence on biologics, ensuring that treatment decisions are increasingly guided by data directly relevant to children rather than adult-derived results. Priority research areas include: evaluating early biologic intervention for potential immune reprogramming and disease prevention and modification; defining pediatric-specific dosing ranges; developing innovative delivery strategies (such as home administration, inhaled biologics and ultra-long-acting formulations); and conducting rigorous cost-effectiveness analyses to assess the feasibility of combination therapies in selected refractory cases. In parallel, broad adoption and validation of standardized quantitative response and remission scores will be essential to guide biologic selection and monitor effectiveness, leading to timely treatment switching or discontinuation.

If successfully implemented, these efforts could progressively redefine the role of biologics in pediatric asthma - from advanced controllers to truly precision-based, patient-centered, disease-modifying therapies.
